# Specific barriers to the conduct of randomised clinical trials on medical devices

**DOI:** 10.1186/s13063-017-2168-0

**Published:** 2017-09-13

**Authors:** Edmund A. M. Neugebauer, Ana Rath, Sunya-Lee Antoine, Michaela Eikermann, Doerthe Seidel, Carsten Koenen, Esther Jacobs, Dawid Pieper, Martine Laville, Séverine Pitel, Cecilia Martinho, Snezana Djurisic, Jacques Demotes-Mainard, Christine Kubiak, Vittorio Bertele, Janus C. Jakobsen, Silvio Garattini, Christian Gluud

**Affiliations:** 1 Brandenburg Medical School Theodor Fontane & Health Services Research Witten/Herdecke University, Campus Neuruppin, Neuruppin, Germany; 2 Orphanet, Institut National de la Santé et de la Recherche Médicale (INSERM), Paris, France; 3 Institute for Research in Operative Medicine, Witten/Herdecke University, Cologne, Germany; 4 Centre de Recherche en Nutrition Humaine Rhone-Alpes, Université de Lyon 1, Hospices Civils de Lyon, Groupement Hospitaler Sud, Pierre Benite, France; 5 QUALISSIMA, 10 Rue Clapier, Marseille, France; 6 AIBILI, Azinhaga de Santa Comba-Celas, Coimbra, Portugal; 7 Copenhagen Trial Unit, Centre for Clinical Intervention Research, Rigshospitalet, Copenhagen University Hospital, Copenhagen, Denmark; 8 European Clinical Research Infrastructure Network (ECRIN), Paris, France; 9 IRCCS Istituto di Ricerche Farmacologiche Mario Negri, Milan, Italy; 10 Department of Cardiology, Holbæk Hospital, Holbæk, Denmark

**Keywords:** Randomised clinical trials, Evidence-based clinical practice, Evidence-based medicine, Assessment, Specific barriers, Medical devices, European Clinical Research Infrastructure Network

## Abstract

**Background:**

Medical devices play an important role in the diagnosis, prevention, treatment and care of diseases. However, compared to pharmaceuticals, there is no rigorous formal regulation for demonstration of benefits and exclusion of harms to patients. The medical device industry argues that the classical evidence hierarchy cannot be applied for medical devices, as randomised clinical trials are impossible to perform. This article aims to identify the barriers for randomised clinical trials on medical devices.

**Methods:**

Systematic literature searches without meta-analysis and internal European Clinical Research Infrastructure Network (ECRIN) communications taking place during face-to-face meetings and telephone conferences from 2013 to 2017 within the context of the ECRIN Integrating Activity (ECRIN-IA) project.

**Results:**

In addition to the barriers that exist for all trials, we identified three major barriers for randomised clinical trials on medical devices, namely: (1) randomisation, including timing of assessment, acceptability, blinding, choice of the comparator group and considerations on the learning curve; (2) difficulties in determining appropriate outcomes; and (3) the lack of scientific advice, regulations and transparency.

**Conclusions:**

The present review offers potential solutions to break down the barriers identified, and argues for applying the randomised clinical trial design when assessing the benefits and harms of medical devices.

**Electronic supplementary material:**

The online version of this article (doi:10.1186/s13063-017-2168-0) contains supplementary material, which is available to authorized users.

## Background

Medical devices (MDs) play an important role in the practice of medicine, with the creativity and diversity of this sector contributing to enhancement in the quality and efficacy of healthcare. MDs cover a wide range of products, from simple bandages to life-supporting devices such as stents, and play a crucial role in the diagnosis, prevention, treatment and care of diseases.

The MD sector has become increasingly important for the healthcare of EU citizens, with an immense influence on expenditure. The MD sector employs approximately 575,000 people in the EU alone, representing over 25,000 companies, of which 95% are small and medium-sized enterprises (SMEs) [1].

While strict regulatory procedures exist for pharmaceuticals, there are no such rigorous regulations for MDs [2]. The accumulating number of scandals, rejections and withdrawals of MDs from the market recently led to the proposal of a Regulation of the European Parliament and of the Council on MDs issued on September 26, 2012; however, the proposal was intensively criticised. Among others, a methodological expert panel of the European Clinical Research Infrastructure Network (ECRIN) requested enforcement of a more rigorous clinical evaluation of MDs regulated by the Medical Device Directive: “[…] *high and medium risk devices (active and inactive implantable medical devices (classes III and IIb) as well as in vitro diagnostic devices need more crucial clinical evaluation before market approval*” [3]. The panel insisted on increasing patient safety through proper scientific assessment of benefits and harms, both in the short and long term, based on results from well-designed randomised clinical trials (RCTs) and other clinical studies: “*More rigorous regulations and sufficient pre-marketing data for high and medium risk medical devices and in vitro diagnostic medical devices are needed not only to increase patient safety, but also to prevent recent medical scandals such as metal-on-metal hip prostheses, stents for intracranial atherosclerotic stenosis, transvaginal meshes, cardiac valves, and cardiovascular implantable electronic devices (pacemakers) that have caused harms and concerns. The pre-marketing assessment and approval of high and medium risk medical devices should be combined with continued post-marketing surveillance to ensure that benefits and harms of device application in real-world settings is similar to existing clinical trial data*” [3]. A supplementary proposal was issued on September 21, 2015, and recently discussed in Luxembourg during a Multi-Stakeholder Workshop of the Joint European Forum for Good Clinical Practice and the MedTech Europe Medical Technology Working Party. Representatives from the MD industry argued that the classical evidence hierarchy cannot be applied for MDs and, thus, that RCTs are not the gold standard for their evaluation. However, given the focus of MD professionals, particularly in SMEs, on the technical aspects of product development, they may not be sufficiently acquainted with evidence-based clinical research. There is a need to build the best evidence on benefits and harms of all interventions adapted to the intrinsic complexity of MDs [4, 5]. To this end, the ECRIN Integrating Activity (ECRIN-IA) project^1^ [6] has identified barriers for good clinical research within trials in general, as well as for trials on rare diseases and nutrition, and assessed how these barriers can be broken down in order to improve their evidence-based clinical practice. The present review is one out of a series of non-interventional systematic reviews aimed to shed light on the current barriers for the conduct of RCTs, as seen from the ECRIN perspective [7–9]. The present review summarises barriers specific to MD trials, and should be viewed in addition to the common barriers to all clinical trials, namely inadequate knowledge and understanding of clinical research and trial methodology, lack of funding, excessive monitoring, restrictive interpretation of privacy law and lack of transparency, overly complex or inadequate regulatory requirements, and inadequate clinical research infrastructures [7], as well as to the barriers to rare disease trials [8]. Barriers to rare disease trials include difficulty in recruiting patients due to their rarity, scattering of patients, limited awareness and knowledge, difficulties achieving accurate diagnosis and identifying patients in health information systems, difficulty in deciding on clinically relevant outcomes, and the need for multi-stakeholder engagement, including that of patients [8]. The specific barriers towards the conduct of RCTs on MDs to be discussed herein include (1) RCT design, (2) minimal requirements for outcome assessment, and (3) regulatory issues specific for MD trials.

## Methods

**Fig. 1:**
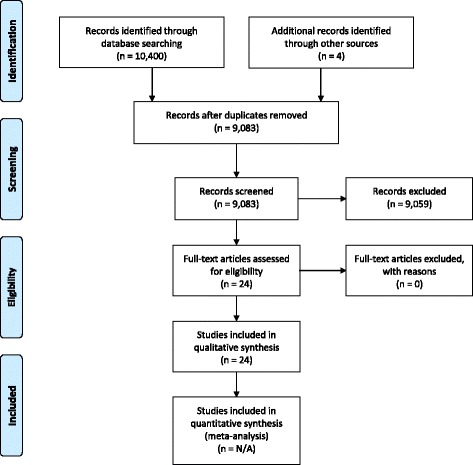
PRISMA flow diagram depicting the selection process of relevant literature

The present review is based on a combination of systematic literature searches and internal ECRIN communications from 2013 to 2017. The systematic literature searches for appropriate articles was performed in May 2016 using The Cochrane Library (Wiley; up until Issue 5, 2016) (including the Cochrane Database of Systematic Reviews, CENTRAL, National Health Service Economic Evaluation Database, and Database of Abstracts of Reviews of Effects (U.S. Library of Medicine)), MEDLINE (Ovid SP; 1946 to May 2016); EMBASE (Ovid SP; 1974 to May 2016) and Science Citation Index Expanded (1900 to May 2016), with search term combinations: “evidence* and (medicine or practice)) or (clinical trial*) or (systematic review*)” plus “barrier* or bottle*neck* or obstacle*” plus “equipment* or suppl* or device”. Articles were screened for relevance in the context of MDs and RCTs. No meta-analyses were performed. Due to the nature of the present review, the results are descriptive and based on conclusions drawn by the ECRIN expert panel. The narrative description of results also poses a limitation of the data collected. The exact search strategy is provided in Additional file 1. A PRISMA flow diagram depicting the selection process and a PRISMA checklist are provided in Fig. 1 and Additional file 2.

## Results and discussion

### Search results

The electronic literature searches resulted in a total of 9079 articles after removal of duplicates. The screening process narrowed the search down to 24 relevant articles listed in Additional file 3. The included articles were overviews and narrative reviews.

### RCTs on MDs

The RCT is the study design reaching the highest level of evidence when assessing the benefits and harms of any given intervention, only surpassed by systematic reviews with meta-analysis of all available RCTs [5]. In many cases, especially when an effective standard procedure exists and can be used as a comparator procedure, randomisation should be possible to perform. In some cases, cluster-randomisation (by clinical centre, hospital, department, etc.) is the most practical and preferable trial design [10].

Bernard et al. [11] recently performed a systematic literature search from January 2000 to July 2012 in order to identify reported problems related to the conduct of conventional RCTs assessing MDs. Based on the discussion by members of the ECRIN-IA methodological expert panel (ECRIN-IA Workshop) and the article by Bernard et al. [11], the authors of the present review believe that at least five perceived and real barriers exist to the conduct of RCTs assessing the benefits and harms of MDs, namely timing of assessment, acceptability, blinding, choice of comparator group and learning curve.

#### Timing of assessment

Choosing the most appropriate time to clinically assess an MD is one specific issue to consider, as MDs often undergo frequent changes in device design after their first introduction in humans. Moreover, as all MDs are systematically subjected to pre-marketing assessment, no newer MD with substantial changes will be allowed onto the market along with older products to unduly compete with them. Older products will only become outdated when any newer device is proven more effective and/or safer. The MD industry is concerned that a trial, which in the best-case scenario takes 2–3 years to complete, will achieve results at a time point where the new device is already outdated. Short timelines for RCTs should therefore be an ultimate goal for clinical investigators that initiate trials on MDs. Moreover, clinical trial units with specific experience in MDs should be authorised to conduct the trials. The ECRIN-IA initiative supports this by providing information on clinical trial units experienced in multinational clinical trials with MDs across Europe [6]. Related to the management of technical changes during the trial phase, device changes should be anticipated during trial planning; changes should be categorised either as ‘substantial’ or ‘not substantial’ [12]. For long-term outcomes, RCTs should be supplemented with registry data. Detailed registries should therefore start in parallel to the clinical trial [13, 14].

The “Idea, Development, Exploration, Assessment, Long-term Follow-up” (IDEAL) collaboration developed a framework for surgical innovation and, more recently, a framework for evaluating and regulating the use of MDs (e.g. implantable devices like pacemakers and hip implants) [15, 16]. The IDEAL consensus group states that extensive preclinical testing is necessary before application of particular implantable devices to patients, and it advocates that a safe regulatory system is implemented for MDs, which does not negatively affect innovation while allowing for appropriate assessment of safety and efficacy [15, 16]. We fully support this. We also support that an assessment should take place before MDs are widely distributed, and that such assessments should be reported fully, irrespective of results.

Another issue of timing is that different levels of experience, for example, of the surgeon or the trial personnel, may lead to different levels of performance when carrying out interventions. An RCT performed too early, before appropriate training and experience has been acquired, may not reflect the true performance of the MD investigated. In such case, an unfavourable assessment could reflect a poorly mastered technique rather than a genuinely ineffective technique. To anticipate such problems, the investigators should try to anticipate how long such learning phases are expected to take, with subsequent splitting of the data into two subgroups – patients randomised during the learning phase compared to those randomised after the learning phase. Conversely, an assessment by an RCT conducted too late could also be problematic if doctors/care providers no longer adhere to a study protocol, or if the recruitment of patients declines dramatically. An example of this is the timing of a previous trial planned to investigate conventional versus laparoscopic cholecystectomy [17]. Initially, there was insufficient support to initiate the trial [17], and while it had been agreed that assessment should be performed in an RCT prior to the experimental intervention being widely distributed, this never became the case. Laparoscopic cholecystectomy was first fully evaluated years after it had been implemented into clinical practice without support from evidence [18, 19]. Unfortunately, the main result of the RCTs conducted years after proved not to be in favour of the laparoscopic approach, by which time it was too late [19]. This is a further argument for the standpoint introduced by Chalmers: “*When new interventions are assessed we should always randomise the first patient*” [20, 21].

#### Acceptability

An issue, which can disrupt patient recruitment and make randomisation with MDs difficult, is when patients and care providers prefer to choose their treatment, and refuse randomisation. This may lead to difficulties in obtaining a sufficient number randomised patients, and lost chances to obtain valid trial results. Further, although surgeons generally like to use new devices, they may be predisposed to believe that the technique they normally use is superior [22]. Only uncertainty (equipoise) related to the best treatment justifies the test of alternative treatments. If surgeons believe that the technique they currently use is the best, they are right in not randomising patients to alternative strategies. However, beliefs ought to be built on evidence, not assumptions. Furthermore, patients may also be misinformed (e.g. through marketing materials by companies) about the potential value of a new device; this may result in reporting bias when patient-reported outcomes are used as outcome measures [17].

Overall, surgeons are more willing to participate in an expertise-based clinical trial than in a conventional trial [22]. A cross-sectional survey showed that 58% of orthopaedic surgeons prefer to participate in expertise-based controlled trials compared to only 17% for conventional RCTs [23]. In addition, there is improved acceptability because surgeons often only perform the procedures that they are used to, which they prefer, and in which they are ‘experts’. Expertise-based RCTs randomise patients to a specialised physician [23], with the advantage of better acceptability, and reduction of execution bias and protocol deviations [23], but the disadvantage of uncertainty of whether the observed difference is related only to the surgeon expertise (enthusiasm bias). Given the fact that a result of a procedure is not only dependant on the surgical expertise, but on a complex interplay between involved parties (surgeon, nurse, team interaction, organisation, etc.), it is important to describe the context in full detail. As a new approach, a qualitative study should be performed before commencing an RCT [24].

#### Blinding

Blinding is an important element in all clinical trials; it reduces measurement bias related to the observer’s, doctor’s or patient’s subjectivity. For ethical or practical reasons, blinding is often more difficult to perform in RCTs on MDs compared to pharmacological RCTs [22]. For non-pharmacological RCTs, alternatives have been developed. Boutron et al. [25] have summarised the different blinding methods used in non-pharmacological trials. Blinding may be complete, partial or only apply to the assessment of outcomes.

When it is not possible to blind healthcare professionals, a blind assessment of the outcome should be planned with experienced and trained staff as outcome assessors. The data managers, the adjudication committee, the independent data monitoring and safety committee, the statisticians, and the conclusion drawers should also be blinded [26–31]. In case blinding is not used, trial authors need to give the reasons for not blinding, and discuss the limitations when reporting the results. As blinding of patients and trial personnel may be less often achievable in some MD trials, objective outcomes must be chosen. The adoption of undisputable objective outcome measures is another strategy for overcoming the limitations of open label trials.

#### Choice of comparator group

The treatment in the comparator group should be selected according to existing standards of medical care, and the best available evidence on existing treatments. It should be common procedure that such selection is based on systematic reviews with meta-analyses showing more benefit than harm [32]. Therefore, one or more systematic reviews with meta-analyses are recommended to select the best alternative. The possibility exists that more than one comparator is suitable, or that different comparators are chosen for specific subgroups, and thus, authors should state the reasons for choice of comparator group. It is often valid to conduct an RCT in which the experimental intervention is added to a standard treatment also used in the control group. Problems concerning patient acceptability and recruitment may arise when the comparator is either an invasive technique or a technique already widely known [11]. From an ethical point of view, it may be inappropriate to offer patients an invasive sham procedure. The more invasive the procedure, the harder it is to justify exposing patients in the control group to risks that may be substantial without any expected benefit. However, it should be emphasised that ethical considerations also apply to treatments received by future patients. The widespread use of unassessed interventions is definitely not ethical. This means that it is important to make participants aware of the overall benefit of the trial, as it is to standardise the context of the trial with regards to preoperative care (of patients or equipment), perioperative care (duration of procedure, instruments, manipulation, or care), and postoperative care and rehabilitation.

#### Learning curve

A particular feature of health technologies using MDs is that the surgeon’s experience and expertise often affect the results of the technique [11, 33]. Different levels of a surgeon’s experience and expertise may lead to different levels of performance when carrying out interventions. A lack of experience may influence the result of the study, penalising the new treatment tested. Many reports indicate that operations performed while the healthcare providers are in a learning phase are associated with greater risks and adverse events compared to operations performed after training has been completed [22, 34]. The learning curve for surgeons and trial personnel should be considered when assessing surgical or interventional techniques. During the development of a new MD, provision must be made for training and learning plans, i.e. minimum expertise for healthcare providers should be defined before they can participate in the trial, experience and knowledge of all healthcare providers should be checked (and improved) before and during the trial, and an ‘expertise-based’ trial should be favoured. Moreover, the use of a new device should be approved before the device is distributed, preferably starting with the already trained trial personnel [15]. To successfully evaluate a MD, the prior experience of the personnel under training should be quantified, the case mix and complexity should ideally be constant, the level of supervision received should be fully described and, ideally, a learning plateau should reach a predefined competency level [22, 33]. Learning should be part of the overall test because it could enable us to reassure patients that the new MD is effective and safe regardless of the level of experience of the healthcare provider. This would add to the external validity of the trial. Moreover, any patient should be randomised whatever the learning phase – the less experience a surgeon has, the greater the uncertainty about the outcome of the test, and therefore the greater the need for randomisation.

### Outcome assessment for trials on MDs

Defining relevant outcomes for clinical trials on MDs is complex. This is partly due to the great variation in complexity and application for the different types of MDs such as pacemakers, insulin pumps, operating room monitors, defibrillators and surgical instruments, and partly due to a large variety of potentially relevant outcomes.

A barrier specifically related to the MD industry is that a common understanding of the concept of outcomes is missing. In clinical trials with MDs, traditional outcomes such as survival, complication rates or surrogates (biomarkers, imaging techniques and omics) are used instead of the more appropriate hermeneutic outcome measures such as quality of life, autonomy, discomfort, disability, and life satisfaction. This does not mean to exclude specific outcomes for the functionality of MDs such as device failure, device breaking, device slipping, migrating of the device or screw loosening, etc. It is important to understand that a hermeneutic outcome measure is a concept, not just a term with mechanical definition. Outcome scales are always aggregate variables rather than individual variables. These variables should be determined in trial design through consensus between patients, doctors and society.

The problem of industry bias on outcome reporting is very relevant to the field of MDs [35, 36]. Trials on MDs funded by industry are prone to report positive outcomes, and to conclude in favour of experimental interventions when obtaining non-significant test results [35, 36]. While industry involvement is necessary to improve technology and to drive innovation of MDs, it must be based on scientific grounds and fully transparent [37].

The methodological expert panel for MDs from ECRIN recently analysed the current common procedures for Health Technology Assessment (HTA) in defining outcomes for MD trials (see http://outcome-measure.ecrin.org/). The HTA institutions emphasise that patient-reported outcomes play an important role in each disease context. Patient-reported outcomes are defined as “*any report of the status of a patient’s health condition that comes directly from the patient, without interpretation of the patient’s response by a clinician or anyone else*” [38]. Outcomes selected to reflect the patient perspective are recommended to include patient satisfaction, patient preference, compliance and acceptance [39]. Furthermore, consequences for the patient’s family and caregivers are considered important. The ECRIN panel concluded that no concrete formulations on the approach of defining appropriate outcome measures for MD trials exist. The ECRIN panel further concluded that the approaches are inhomogeneous among HTA institutions. It also stated that separating MD assessment from the procedure is not possible in many cases, which makes it difficult to identify the assignable cause of effect [40]. Thus, the outcome measures selected for MDs should reflect the whole procedure, and all different kinds of settings that the MD can be used in. According to the expert panel, the choice for the most appropriate outcome measure depends on the (1) primary objective (increase of benefit, reduction of harm); (2) state of development of the technology (feasibility, effectiveness, efficiency); (3) quality criteria (validity, reliability); (4) acceptance (by patients, physicians and scientific community); and (5) acknowledgement of the value of better tolerability or convenience.

Support for the definition of relevant outcome measures is provided by several initiatives, e.g. initiatives which establish core outcome sets with a focus on minimal requirements for data collection. These include outcome measures, which are agreed on and defined as necessary to report in clinical trials (e.g. the EU-funded Core Outcome Measures in Effectiveness Trials Initiative (http://www.comet-initiative.org)). Initiatives like this are engaged to bring researchers together to develop core outcome sets related to many procedures and diseases along with corresponding stakeholders, e.g. patient and industry representatives. Specific for MDs, ECRIN has developed an MD outcome measure database available online (http://outcome-measure.ecrin.org/). This database supports researchers involved in clinical trials and HTAs on MDs by providing a comprehensive view of outcome measures selected from HTAs, systematic reviews and horizon scans published after 2008. The database reflects commonly used outcome measures of international institutions involved in HTAs, such as members of the International Network of Agencies for Health Technology Assessment, EUnetHTA, and non-profit members of Health Technology Assessment International [41]. The utilisation of defined core outcome measures in combination with the information provided by the ECRIN MD outcome measure database will provide further support in the selection of relevant outcome measures, and therewith increase comparability between trials [42]. For further information see: http://www.proqolid.org and http://www.mapi-trust.org.

### Lack of scientific advice, regulations and transparency

After more than 3 years of discussion of the proposal for a Regulation of the European Parliament and of the Council on Medical Devices an agreement is expected in 2019 [43]. In a Multi-Stakeholder Workshop of the Joint European Forum for Good Clinical Practice and the MedTech Europe Medical Technology Working Party in Luxembourg in October 2015, regulatory barriers to the conduct of trials on MDs were identified and recommendations for improvements discussed. Key points are presented below.

#### Early scientific advice and expert panels

The medical technology industry is dominated by large numbers of SMEs. They are not trained in running trials or in trial methodology, but have a high output of diverse and innovative products.

Access to early scientific advice, especially for smaller companies and academia, needs to be as easy and affordable as possible. Early scientific advice about the clinical development strategy and RCTs for their devices is wished for.

#### Regulatory and ethical requirements

The new Regulation on Medical Devices will impose increased responsibilities and well-defined interactions between all economic parties involved, like MD manufacturers, authorised representatives, importers and distributors. Many of Europe’s medical technology companies are lacking the infrastructure to fully deal with their obligations.

To help address some of the regulatory and ethical challenges regarding MD trials, ECRIN has developed a central resource for information about clinical trial regulatory and ethical requirements covering over 22 European countries, called the CAMPUS database (http://campus.ecrin.org/). CAMPUS covers multiple study types, including clinical investigations of MDs, drug-device combination studies and nutritional studies. Toolkits have been prepared for various countries to provide country-specific information on regulatory and ethical requirements in MD studies. For each country for which they have been prepared, these toolkits include the definition and legal basis for MD studies, information on insurance, sponsors, investigators, competent authorities, ethics committees, data protection and healthy volunteers/patients, and specific country requirements, if any, regarding issues such as registries, monitoring and informed consent (http://campus.ecrin.org/).

#### Transparency

Trial registration increases transparency and is a standard requirement in the field of medicinal trials. Trial registration should be required for MD trials too [5, 37, 44]. Similarly, the need to make trial data available for secondary analysis is being adopted by medical journals and should also be adopted in MD trials [5, 37, 44]. There is still a significant lack of transparency regarding trial protocols, processes, data management, statistical analysis plans and meta-data as well as results and de-identifiable individual patient data from MD trials all trials in general [2, 37]. For patient safety and public control, the publication of all information on the process and the basis for approvals of MDs, as well as in vitro diagnostic devices on a publicly accessible website irrespective of risk classes, is demanded by more and more parties [37, 44]. The industry stakeholders argue with the patent law in hand, and claim that the intellectual rights to the MD are no longer secured. After a long debate, the members of the Multi-Stakeholder Workshop recommended considering greater transparency around clinical evaluation of MDs in general and regarding the decisions that notified bodies make when reviewing high-risk devices in particular. Under the current Council text on the Regulation, the study protocol and a summary of the results are to be made publicly available in European databases for clinical trials [37, 43]. Moreover, access to individual patient data should also be secured [37, 43].

Herein, we have identified three overall embracing barriers to the conduct of RCTs on MDs, encompassing: (1) when and how to conduct RCTs, (2) requirements for outcome assessment, and (3) regulatory issues specific for MD trials (Table 1). These barriers should be seen as additions to the barriers that exist for all RCTs (inadequate knowledge and understanding of clinical research and trial methodology, lack of funding, excessive monitoring, restrictive interpretation of privacy law and lack of transparency, overly complex or inadequate regulatory requirements, and inadequate clinical research infrastructures) as well as the barriers to the conduct of RCTs on rare diseases (difficulty recruiting patients as a direct consequence of rarity, scattering of patients, limited awareness and knowledge, difficulties achieving accurate diagnosis and identifying patients in health information systems, difficulty deciding on clinically relevant outcomes, and the need for multi-stakeholder engagement, including that of patients, for successful clinical research) [7–9].

**Table 1:** Perceived or actual barriers to the conduct of randomised clinical trials (RCTs) within medical devices (MDs), and potential solutions

Perceived or actual barriers to the conduct of RCTs within MDs:	Potential solutions and counter arguments
Timing of the assessment	Extensive preclinical testing is necessary before application to the first patient. Short timelines necessary. The Idea, Development, Exploration, Assessment, Long-term follow-up (IDEAL) collaboration developed a framework for different study types for the different stages of surgical innovation, which is also supported by the members of the methodological expert panel. By any means, an assessment should take place before an MD is widely distributed.
Acceptability	One possibility is an expertise-based randomised clinical trial. The advantages are better acceptability and reduction of execution bias and protocol deviations.
Blinding in MD trials difficult	Blinding may be complete, partial or only apply to the assessment of endpoints. Blinding of outcome assessors should be possible in most studies and should be used as a standard procedure whenever possible – objective endpoints should be preferably adopted.
Comparator in MD trials	Treatment in comparator arms should be selected per existing standards of medical care and best-available evidence on existing treatments. The possibility that more than one comparator is appropriate or different comparators exist in specific subgroups should be considered.
Learning curve	The learning phase must be considered in the trial so that any benefit provided by the device or health technology can be evaluated accurately. Trainee’s prior experience should be quantified, the case mix and complexity should ideally be constant and the level of supervision received should be fully described.
Minimal requirements for outcome assessment	A common understanding on the concept of outcomes is missing in the MD industry. The outcomes included in the analysis should reflect the whole procedure and all different kinds of settings the MD can be used in. For MDs, the MD outcome measure database was developed by the European Clinical Research Infrastructure Network (ECRIN) and is now online. For long-term outcomes, RCTs should be supplemented by parallel prospective registry data. Registry-based RCTs should be considered.
Transparency	In the current Regulation, the study protocol and a summary of the results are to be made publicly available in European databases for clinical trials.
Early scientific advice and expert panels	Early scientific advice with regard to the clinical development strategy and clinical studies for their devices is proposed.
Information about clinical trial regulatory and ethical requirements	Country-specific information on regulatory and ethical requirements in MD studies are available.

MDs range from bandages to life support machines, and MDs are classified in the EU in four risk categories from low to high risk [45]. The risk associated with the MD depends on the duration of contact with the body, invasiveness and whether it has a local or a systemic effect. Medium- and high-risk devices must be certified by one of the notified bodies, organisations that are accredited to assess a product’s compliance with EU legislation (CE mark). Rejections and withdrawals of MDs due to serious adverse effects with harm to the patients, however, have led to the request to amend the European regulation process, which is currently ongoing. A recent paper deals with the problem of dissemination bias in clinical research, and concludes that the issue has not been resolved despite several initiatives to reduce it [46].

## Conclusions

The authors of the present review, including the expert panel from ECRIN responsible for the field of MDs, were faced with serious methodological challenges and arguments not to run RCTs for different reasons. This article concentrates on the discussion of the most important specific barriers for RCTs with MDs and offers potential solutions and counter arguments favouring RCTs at a certain point of development.

We propose to follow the recommendations for the assessment of surgery based on a five-stage description of the surgical development process [15]. RCTs should be used whenever possible to investigate efficacy. Difficulties in performing RCTs should be addressed by measures to evaluate learning curves and alleviate equipoise problems. Other types of experimental designs should be used when RCTs are not feasible [11]. We need to increase the truthfulness in published clinical research, and to publish all clinical research results [5, 30, 31, 37, 47–51].

## Additional files

Additional file 1: Literature search strategy. Exact search strategy applied for analyses. (DOCX 13 kb)

Additional file 2: PRISMA 2009 checklist. (DOC 63 kb)

Additional file 3: Relevant references from literature search. Results listed from literature search in the form of relevant publications. (DOCX 18 kb)

## Abbreviations

COMET: Core Outcome Measures in Effectiveness Trials Initiative
ECRIN: European Clinical Research Infrastructure Network
ECRIN-IA: ECRIN-Integrating Activity
HTA: health technology assessment
IDEAL: Idea, Development, Exploration, Assessment, Long-term Follow-up
MD: medical device
RCT: randomised clinical trial
SME: small- and medium-sized enterprises

## Footnotes

1 Funded by the European Union Framework Programme 7 (EU FP7; grant agreement no. 284395), ECRIN-IA involved 23 countries and brought together diverse stakeholders to overcome barriers to clinical research in three particularly difficult areas (rare diseases, medical devices and nutrition). Specifically, the project aimed to develop tools, services and infrastructure to facilitate multinational clinical research in Europe, and to support the development of pan-European disease networks to drive clinical projects. This, in turn, was intended to improve Europe’s attractiveness to industry, boost its scientific competitiveness and result in better healthcare for European citizens. Originally planned for 4 years (2012 to 2015), the clinical trials work package was extended until 2017.
