# Supplementary material for: Specific barriers to the conduct of randomised clinical trials on medical devices
Source: Trials. 2017 Sep 13;18:427. doi: 10.1186/s13063-017-2168-0 (PMC5597993; doi:10.1186/s13063-017-2168-0)
Supplement: Supplementary file 3 — Relevant references from literature search. Results listed from literature search in the form of relevant publications. (DOCX 18 kb) [file 13063_2017_2168_MOESM3_ESM.docx]

Additional file 3. Relevant references from literature search (n=24)

- Altenstetter C. EU and member state medical devices regulation. International Journal of Technology Assessment in Health Care. 2003;19(1):228-48.
- Bucci VA, Reiss JB, Hall NC. New obstacles in the path of marketing new medical devices. Journal of Health Care Technology. 1985;2(2):81-96.
- Burridge JH, Hughes AM. Potential for new technologies in clinical practice. Current Opinion in Neurology. 2010;23(6):671-7.
- Demotes-Mainard J. [ECRIN (European clinical research infrastructures network), a pan-European infrastructure for clinical research]. [French]. Bulletin de l'Academie Nationale de Medecine. 2010;194(9):1683-94.
- Demotes-Mainard J. Clinical research networks as a support to independent clinical trials. Clinical Therapeutics. 2015;Conference: 12th Congress of the European Association for Clinical Pharmacology and Therapeutics, EACPT 2015 Madrid Spain.
- Diaconu K, Chen YF, Manaseki-Holland S, Cummins C, Lilford R. Medical device procurement in low- and middle-income settings: protocol for a systematic review. Systems Review. 2014;3:118.
- Krucoff MW, Brindis RG, Hodgson PK, Mack MJ, Holmes DR. Medical Device Innovation: Prospective Solutions for an Ecosystem in Crisis Adding a Professional Society Perspective. JACC Cardiovascular Interventions. 2012;5(7):790-6.
- Lockwood C, Marinoni G, Gallo P, Kowal S. Inpatient reimbursement landscape for medical device and diagnostics in developed markets. Value in Health. 2012;Conference: 17th Annual International Meeting of the International Society for Pharmacoeconomics and Outcomes Research, ISPOR 2012 Washington, DC United States.
- Marcus HJ, Payne CJ, Hughes-Hallett A, Marcus AP, Yang GZ, Darzi A, et al. Regulatory approval of new medical devices: cross sectional study. BMJ. 2016;353:i2587. Epub 2016/05/22.
- Morgan JM, American Heart A, North American Society of P, Electrophysiology. New device indications: practice and cost implications in Europe. Cardiac Electrophysiology Review. 2003;7(1):49-53.
- Morscher EW. Failures and successes in total hip replacement--why good ideas may not work. Scandinavian Journal of Surgery: SJS. 2003;92(2):113-20.
- Normand SL, Marinac-Dabic D, Sedrakyan A, Kaczmarek R. Rethinking analytical strategies for surveillance of medical devices: the case of hip arthroplasty. Medical Care. 2010;48(6 Suppl):S58-67. Epub 2010/05/18.
- Pieper D, Neugebauer EAM. Das geht so nicht! -Chirurgische Innovationen brauchen die Überprüfung in kontrollierten klinischen Studien. Der Chirurg [Submitted]. 2016.
- Polisena J, Gagliardi A, Urbach D, Clifford T, Fiander M. Factors that influence the recognition, reporting and resolution of incidents related to medical devices and other healthcare technologies: a systematic review. Systems Review. 2015;4:37.
- Portner PM. Economics of devices. Annals of Thoracic Surgery. 2001;71(3 Suppl):S199-201; discussion S3-4.
- Robinson EJ, Kerr CEP, Stevens AJ, Lilford RJ, Braunholtz DA, Edwards SJ, et al. Lay Public's Understanding of Equipoise and Randomisation in Randomised Controlled Trials. Health Technology Assessment. 2005;9(8).
- Rose EA, Moskowitz AJ, Packer M, Sollano JA, Williams DL, Tierney AR, et al. The rematch trial: Rationale, design, and end points. Annals of Thoracic Surgery. 1999;67(3):723-30.
- Sedrakyan A, Campbell B, Merino JG, Kuntz R, Hirst A, McCulloch P. IDEAL-D: a rational framework for evaluating and regulating the use of medical devices. BMJ. 2016;353:i2372. Epub 2016/06/11.
- Shah SG, Robinson I. Benefits of and barriers to involving users in medical device technology development and evaluation. International Journal of Technology Assessment in Health Care. 2007;23(1):131-7.
- Slottow TL, Steinberg DH, Waksman R. Overview of the 2007 Food and Drug Administration Circulatory System Devices Panel meeting on patent foramen ovale closure devices. Circulation. 2007;116(6):677-82.
- Waetjen LE, Parvataneni R, Varon S, Saberi NS, Jacoby VL, University of California Fibroid N. Obstacles to Studying Emerging Technologies. Obstetrics and Gynecology. 2015;126(2):391-5.
- Weber S, Haverich A. [Pioneering surgical innovations in Germany: Part 2: public funding and origins of surgical innovations]. Der Chirurg; Zeitschrift fur alle Gebiete der Operativen Medizen. 2016;87(5):433-7.
- Weber S, Haverich A. [Pioneering surgical innovations in Germany: Part 1: generation of medical evidence]. Der Chirurg; Zeitschrift fur alle Gebiete der Operativen Medizen. 2016;87(5):423-32.
- Wente MN. Barriers to clinical studies involving medical devices. [German]. Zeitschrift fur Evidenz, Fortbildung und Qualitat im Gesundheitswesen. 2012;106(5):315-9.
